# Basalt Fiber Modified Ethylene Vinyl Acetate/Magnesium Hydroxide Composites with Balanced Flame Retardancy and Improved Mechanical Properties

**DOI:** 10.3390/polym12092107

**Published:** 2020-09-16

**Authors:** Dongwei Yao, Guangzhong Yin, Qingqing Bi, Xu Yin, Na Wang, De-Yi Wang

**Affiliations:** 1 Sino-Spanish Advanced Materials Institute, Shenyang University of Chemical Technology, Shenyang 110142, China; ydw18100331868@163.com (D.Y.); biqingqing20200909@163.com (Q.B.); 18240119126@163.com (X.Y.); 2 IMDEA Materials Institute, C/Eric Kandel, 2, Getafe, 28906 Madrid, Spain; guangzhong.yin@imdea.org

**Keywords:** ethylene vinyl acetate (EVA), magnesium hydroxide (MH), basalt fiber (BF), flame retardancy, mechanical properties

## Abstract

In this study, we selected basalt fiber (BF) as a functional filler to improve the mechanical properties of ethylene vinyl acetate (EVA)-based flame retardant materials. Firstly, BF was modified by grafting γ-aminopropyl triethoxysilane (KH550). Fourier transform infrared spectroscopy (FTIR), thermogravimetric analysis (TGA), scanning electron microscope (SEM), and energy dispersive X-ray spectroscopy (EDS) were used to comprehensively prove the successful modification of the BF surface. Subsequently, the modified BF was introduced into the EVA/magnesium hydroxide (MH) composites by melt blending. The limiting oxygen index (LOI), UL-94, cone calorimeter test, tensile test, and non-notched impact test were utilized to characterize both the flame retardant properties and mechanical properties of the EVA/MH composites. It was found that the mechanical properties were significantly enhanced without reducing the flame retardant properties of the EVA/MH composites. Notably, the surface treatment with silane is a simple and low-cost method for BF surface modification and the pathway designed in this study can be both practical and effective for polymer performance enhancement.

## 1. Introduction

Ethylene vinyl acetate (EVA) copolymer with different contents of vinyl acetate is widely used in various industries. It is widely used in the cable industry due to its advantages of softness, high filling capacity, strong processing ability, good environmental stress cracking resistance, and heat resistance [1,2]. However, EVA resin is easy to burn and releases a lot of smoke during burning. Therefore, it is necessary to improve the flame retardancy. Flame retardancy can be achieved by adding flame retardants, e.g., aluminum trihydroxide (ATH) [3], layered double hydroxides (LDHs) [4], magnesium hydroxide (MH) [5], etc., among which MH is an environment-friendly flame retardant because of its decomposition temperature (about 340 °C, being higher than that of aluminum hydroxide), smoke suppressibility, non-toxicity, and wide use in halogen-free polymer materials. However, due to its high interface energy and strong hydrophilicity, the compatibility between MH and polymer matrix is poor, and the mechanical properties of EVA are decreased in accordance with the addition of MH [6,7].

Fibers that are characterized by low specific gravity, high specific strength, and specific modulus, are widely used to reinforce material, and can effectively improve the mechanical properties of the composites [8]. Common fibers mainly include glass fiber (GF) [9], carbon fiber (CF) [10] and basalt fiber (BF) [11], etc. Among them basalt fiber is a type of mineral fiber, made of basalt. BF has many excellent properties, such as good modulus, good stability, good chemical resistance, high mechanical strength, high temperature resistance, and convenient preparation [12]. BF is similar to GF, and has better physical properties than GF, and obviously a lower price than carbon fiber [13,14,15,16,17]. However, BF is inorganic in nature and therefore has a poor interface interaction with polymer matrix. Therefore, a surface treatment of BF is essential, and many related studies have been reported. For examples, Attia et al. [18] synthesized an organic polyaniline directly on the surface of BF and formed an organic polymer shell on the surface of the inorganic fiber—the tensile strength property was enhanced by 38% and 53% compared to blank and unmodified basalts, respectively. Guo et al. [19] treated BF by using the economical silane agent γ-aminopropyl triethoxysilane (KH550) as a modifier, which showed 194.12% enhancement in impact strength compared to that of pure polypropylene (PP). Zhang et al. [20] modified BF by grafting organic/inorganic composites, and attaching a biofilm rich in microorganisms for wastewater treatment. The results showed that the adhesion rate, immobilization ratio, and biomass of modified BF were higher than that of BF. In Wang’s work [21], a continuous and compact graphene oxide (GO) layer was grafted onto the surface of BF using biomimetic polydopamine as a bridge to improve the mechanical and tribological properties of polyamide 6 (PA6). The impact and flexural strength of the PA6 composites indicated that the introduction of GO gave a large improvement in interface bonding performance between BF and PA6 matrix.

In this work, we used KH550 to modify the surface of BF and introduced the modified basalt fiber into the flame retardant EVA/MH composites matrix. Furthermore, we also studied the effect of BF filler and EVA/MH composites on the flame retardancy and mechanical properties of the composites. It was expected that the mechanical properties of the new composites would be enhanced. The fire safety performance of the composites after fiber introduction was also systematically considered.

## 2. Materials and Methods

### 2.1. Materials

Ethylene vinyl acetate (EVA, UL00628) was supplied by Shandong Lianhong New Materials Co., Ltd. (Shandong, China); Magnesium hydroxide (MH) was obtained from Liaoning Jinghua New Material Co., Ltd. (Liaoning, China). Basalt fiber (BF, 3 mm in length) was supplied by Anjie Composites Co., Ltd. (Zhejiang, China). γ-Aminopropyl triethoxysilane (KH550) was provided by Nanjing Lian Si Chemical Co., Ltd. (Jiangsu, China). Anhydrous ethanol was purchased from Tianjin Damao Chemical Reagent Factory. (Tianjin, China). All chemicals were of analytical grade purity and used without any further purification.

### 2.2. Pretreatment of Basalt Fiber

In order to remove the impurities on the surface of the basalt fiber and the wetting agent coated on the surface during the production process, we adopted the muffle furnace burning method to obtain the pretreated basalt fiber. An appropriate amount of BF was heated from room temperature to 350 °C over 30 min and held for 2 h in muffle. When the muffle furnace temperature fell below 200 °C, the BF was taken out and cooled to room temperature giving rise to pretreated basalt fiber (PBF).

### 2.3. Surface Modification of Basalt Fiber

**Scheme 1:**
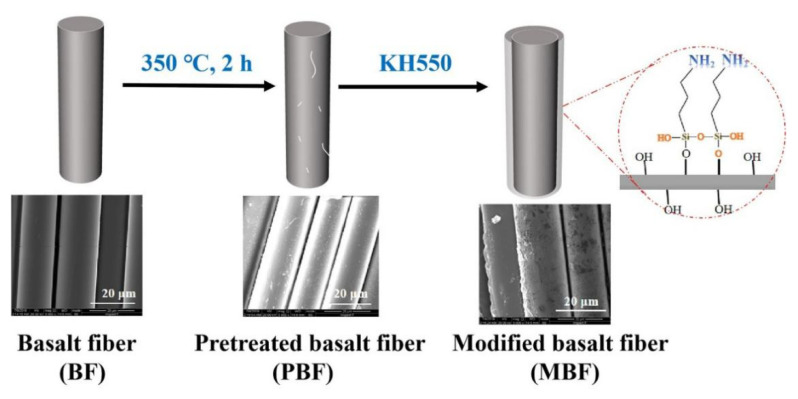
Preparation of modified basalt fiber.

The surface treatment of BF was performed according to the literature [15,22]. First, PBF was immersed in the silane coupling agent (KH550, 0.75 wt.%) in water/ethanol (w/w, 20/1) for 60 min. After that, the fibers were reacted and dried in a vacuum oven at 120 °C for 2 h. Subsequently, the fibers were washed five times with deionized water and placed in an oven at 80 °C for 24 h. The silane-treated fibers were marked as modified basalt fiber (MBF). The schematic illustration is displayed in Scheme 1.

### 2.4. Fabrication of EVA/MH-BF Composites

EVA, MH and BF were mixed according to the formulations in Table 1, and both the front roller and back roller were set to 100 °C to obtain EVA/MH-BF composites.

### 2.5. Characterization

Fourier transform infrared spectra (FTIR) were obtained on a Nicolet MNGNA-IR560 (Artisan Technology Group, Austin, TX, USA) with range from 4000 cm^−1^ and 400 cm^−1^ and used the attenuated total reflectance method (ATR).

Thermogravimetric analyses (TGA) was tested on a STA 449C thermal analyzer (Selb, Germany) from 40 to 800 °C at a heating rate of 10 °C/min under nitrogen atmosphere.

The surface morphology of the basalt fiber and carbon residue after the cone calorimeter test was observed by a scanning electron microscope (SEM) (FEI, inspection F50, USA) under 20 kV accelerating voltage and 8 mm working distance between probe and sample surface. The specimens were sputter coated with gold prior to the measurements, and the surface of the basalt fiber was analyzed by energy dispersive X-ray (EDX).

The limiting oxygen index (LOI) value was measured using an SS-1005 oxygen index instrument (Taiwan Songshu Testing Instrument Co., Ltd., Taiwan, China) according to ASTM D2863-2013 with a sheet dimension of 130 mm × 6.5 mm × 3.2 mm.

The vertical burning test was performed by an SS-3001 burning chamber (UL-94, Taiwan Songshu Testing Instrument Co., Ltd., Taiwan, China) with the sample dimensions of 127 mm × 13 mm × 3.2 mm according to ASTMD3801-2010 standard.

The cone calorimeter tests were conducted on a cone calorimeter (FTT, England, United Kingdom), following the ISO 5660-1:2015 standard procedures. Squared specimens (100 mm × 100 mm × 4 mm) were horizontally irradiated at a heat flux of 50 kW/m^2^, corresponding to a medium fire scenario. At least 2 specimens were measured to obtain the average values.

The tensile test was carried out on the tensile testing machine (9250HV, Instron Engineering Corporation, USA), according to ISO 37/2017, and the average values of three samples in each group were taken. The impact test of unnotched splines (80 mm × 10 mm × 4 mm) was carried out on an impact testing machine (ISO 2932003). The average value of five samples in each group was taken, and the impact span was 60 mm.

## 3. Results and Discussion

### 3.1. Structural Characterization of Modified Basalt fiber

**Figure 1:**
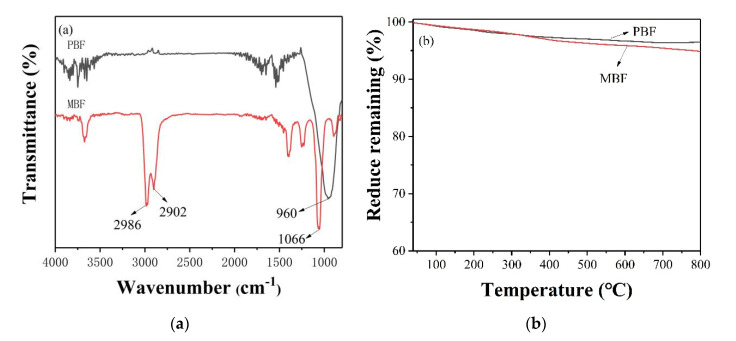
(**a**) Fourier transform infrared spectra (FTIR) of the pretreated basalt fiber (PBF) and modified basalt fiber (MBF); (**b**) Thermogravimetric analysis (TGA) curves of PBF and MBF under N_2_ atmosphere.

FTIR (Figure 1a) and TGA (Figure 1b) were used to detect the functional groups on the BF surface. As can be seen in Figure 1a, there is an absorption peak at 960 cm^−1^ on the FTIR curves of the PBF, which can be attributed to Si–OH. The absorption peak at 1066 cm^−1^ corresponds to the Si–O–Si bond of KH550 grafted BF (Scheme 1) [23]. Peaks at 2986 cm^−1^ and 2902 cm^−1^ are assigned to be antisymmetric and symmetric tensile vibration of the –CH_2_– group in KH550, respectively [24]. The TGA curves of PBF and KH550 modified basalt fibers are shown in Figure 1b. It can be inferred that the weight loss rate of PBF is mainly due to the loss of water. The degradation of KH550 MBF is faster than that of PBF, which may be due to the dehydration of silicone alcohol and the degradation of silane coupling agent [25]. Both FTIR and TGA results confirm the successful introduction of KH550 onto the BF surface.

**Figure 2:**
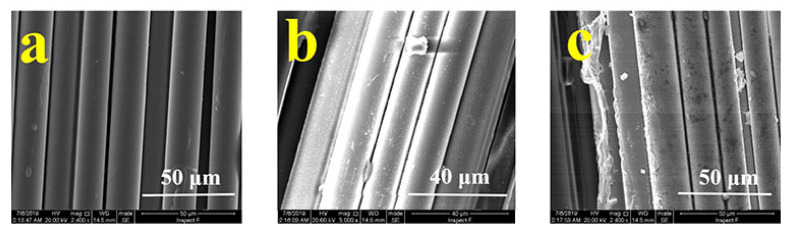
Scanning electron microscope (SEM) images of (**a**) basalt fiber (BF), (**b**) PBF and (**c**) MBF.

Furthermore, SEM was used to study the surface morphological changes of basalt fibers after surface modification. In order to reduce fiber fracture in the process of drawing, BF is usually accompanied by a layer of wetting agent, which makes its surface smooth and decreases the compatibility with the matrix accordingly [26] (Figure 2a). We can see the surface of the PBF is significantly rougher than that of BF (Figure 2b), which is due to the removal of organic matter on the surface of the basalt fiber. Figure 2c shows scanning images of MBF. Obviously, the roughness of the surface of the basalt fiber increased significantly at this time, which directly proves the successful surface modification.

**Figure 3:**
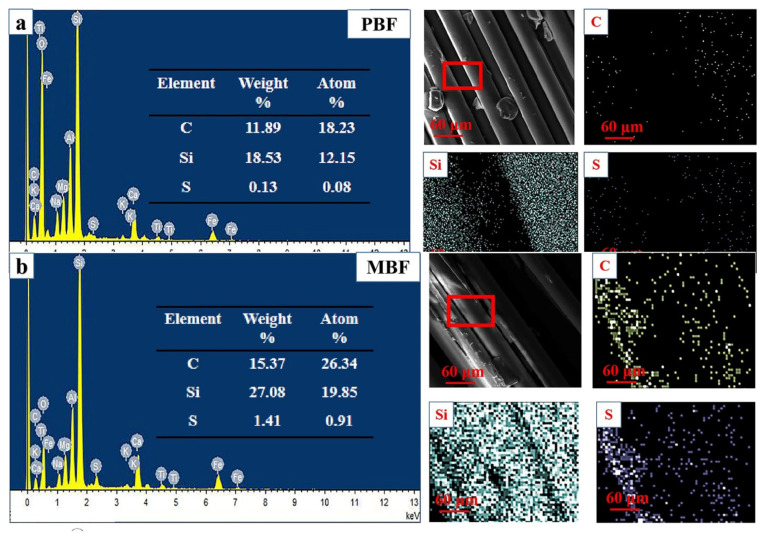
Energy dispersive X-ray (EDX) data, SEM images and the corresponding element mapping for (**a**) PBF and (**b**) MBF.

Then EDX and EDX mapping test were carried out for both PBF (Figure 3a) and MBF (Figure 3b). To reduce the test error, the fibers were immersed in mercaptan solution for 24 h, and S element (with relative high sensitivity) was used instead of N element. It can be seen from Figure 3 that the content of S element increases significantly, which means that the N element introduced by the –NH_2_ group of KH550 on the MBF surface increases.

### 3.2. Flame Retardant Properties

**Figure 4:**
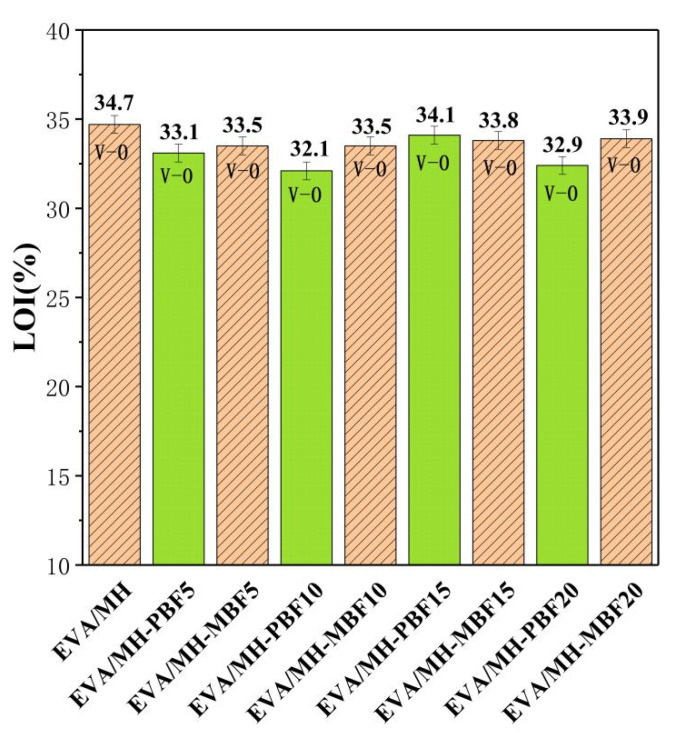
Limiting oxygen index (LOI) and UL-94 results for ethylene vinyl acetate/magnesium hydroxide-basalt fiber (EVA/MH-BF) composites.

The flame retardancy of composites was evaluated via LOI and UL-94 first. Figure 4 shows the LOI and UL-94 results of EVA/MH-BF composites. The LOI value of flame retardant EVA/MH is 34.7%, and the rate of UL-94 is V-0. The fibers during the combustion process produce a wicking effect, [27] which slightly reduces the LOI to a certain extent (Figure 4). Fortunately, The LOI values of all the EVA/MH composites with basalt fiber were decreased less than 3%. It can be further seen that almost all the LOI values of MBF-added EVA/MH composites are slightly higher than that of PBF-added EVA/MH composites with same fiber content, indicating KH550 modification of BF has an obviously positive effect on EVA/MH composites. Meanwhile, the same situation occurs in the mechanical properties section, which is discussed later. Notably, all samples reach V-0 rating, proving that the existence of basalt fiber does not reduce its fire protection grade.

**Figure 5:**
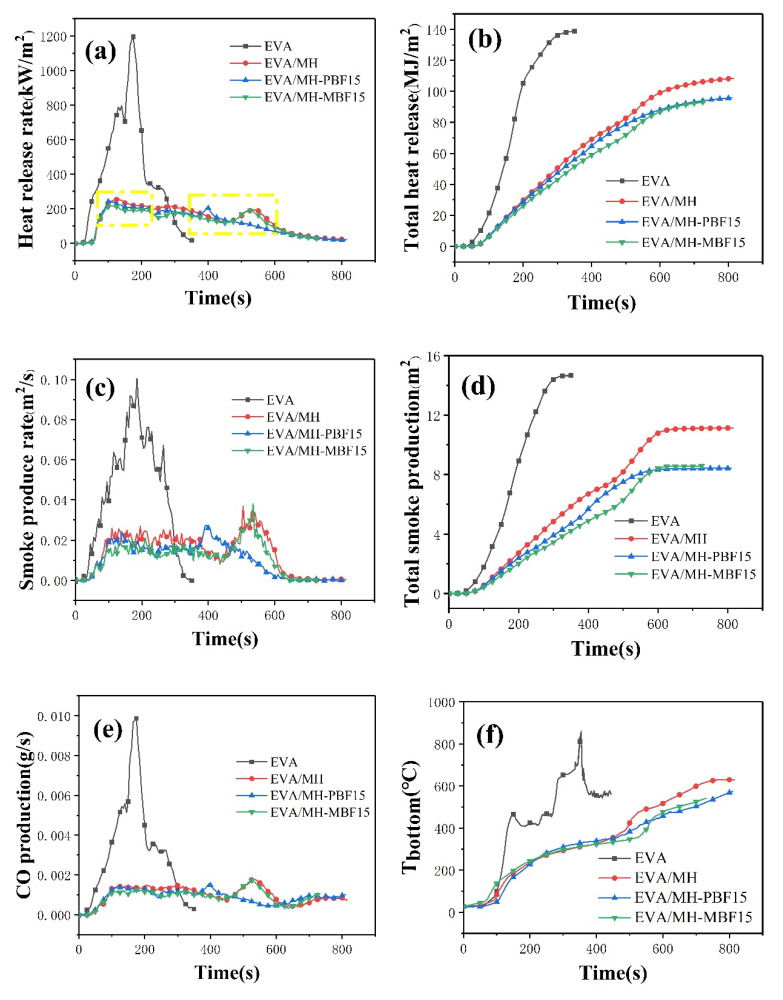
(**a**) heat release rate (HRR), (**b**) total heat release (THR), (**c**) smoke produce rate (SPR), (**d**) total smoke production (TSP), (**e**) CO production rate (COP), (**f**) bottom temperature value of EVA and EVA/MH-BF composites.

The cone calorimetry test was used to measure the combustion performance of EVA and EVA composites. The results of the combustion test are shown in Figure 5, including the heat release rate (HRR), smoke produce rate (SPR), total heat release (THR), total smoke production (TSP), CO production rate (COP), and bottom temperature (Tbottom). While the main characteristic parameters, such as the time to ignition (TTI), the peak of HRR (pHRR), the peak of SPR (pSPR), the average of HRR (AvHRR), total smoke production (TSP), Fire Growth Rate Index (FIGRA), and the time of the peak of HRR (TpHRR) data, are summarized in Table 2.

It can be seen that the EVA/MH-BF curves have a similar trend to the EVA/MH curves and the data are very close in Figure 5a,c,e,f, this indicates that the addition of basalt fiber does not damage the flame retardant properties of EVA/MH composites to a significant extent. As can be seen in Figure 5a, two peaks (as marked in the yellow wireframe) appeared in all the three HRR curves of the composites. Typically, the first peak appears at about 120 s, and the second peak appears at about 540 s. The appearance of the two peaks may be due to the following: increasing oxygen consumption in the initial stage leads to an increase of the heat release rate. With the appearance of the MgO shell in the carbon layer [28], the exothermic rate and the first peak value decrease. However, the fragile carbon layer cannot blockthe heat and oxygen, thus increasing the heat release rate again. Afterwards, the formation of the carbon layer gradually becomes complete, and the heat release rate decreases again until the end of the combustion process.

As shown in Figure 5b,d, the THR and TSP of EVA/MH-BF composites are lower than those of EVA/MH composites. As shown in Table 2, the THR and TSP of EVA/MH composites are 109 MJ/m^2^ and 10.1 m^2^, respectively. Compared with that of EVA/MH composites, the THR values of EVA/MH- PBF15 and EVA/MH-MBF15 composites decreased by 12% and 15%, respectively; and TSP values decreased by 17% and 15%, respectively. The results show that the addition of fiber inhibits the heat and smoke emission of EVA/MH-BF composites, which may be because basalt fiber can connect with the residual carbon to maintain a condensed phase and act as a barrier between the flame and the matrix.

The fire growth rate index (FIGRA) is an important parameter to measure the risk of fire development. We found that FIGRA increases slightly after adding basalt fiber. As listed in Table 2, the FIGRA of EVA/MH, EVA/MH-PBF15, and EVA/MH-MBF15 are 2.00, 2.30, and 2.19 kW/(m^2^·s), respectively. Herein, we assume that the increasing of FIGRA is due to the wick effect [27] of the fiber in the composites.

**Figure 6:**
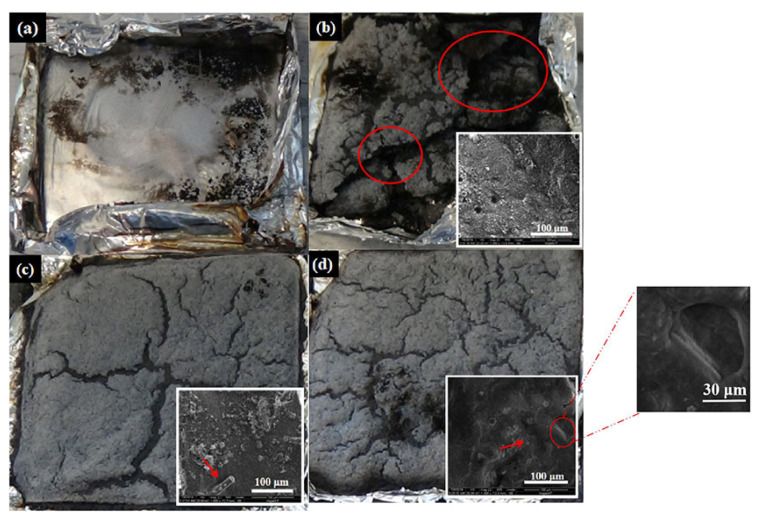
Digital photos and SEM photos of the char residue and its microphotographs of the external surface (**a**–**d**) of (**a**) EVA; (**b**) EVA/MH; (**c**) EVA/MH-PBF15; (**d**) EVA/MH-MBF15.

To clarify the reasons for the intensity of the residue reinforced by BF, the digital photos and SEM photos of EVA, EVA/MH, EVA/MH-PBF15 and EVA/MH-MBF15 after cone test were obtained as shown in Figure 6. As can be seen from Figure 6b–d, the char layer of the EVA composites without BF was fragile and easily collapsed into cracks, while the damage in Figure 6b is the most severe. The broken carbon layer provides a channel for heat and oxygen, causing the combustion intensity to increase again; after the adding of fiber, BF can connect with the carbon residue and ensure that the condensed phase will not be destroyed, so the EVA/MH-BF composites obtain further decreased heat release and total smoke production.

**Figure 7:**
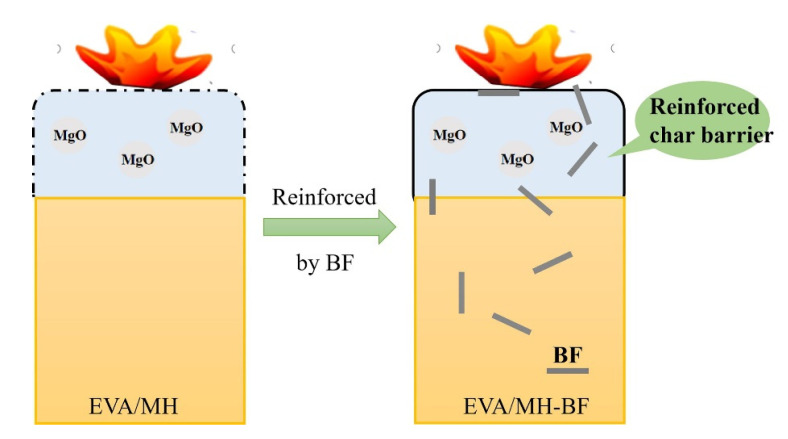
Schematic diagram of combustion of EVA composites.

According to all the discussed results, the effect of BF on the flame retardancy and carbon residue of EVA/MH composites can be summarized as shown in Figure 7. The carbon layer formed by the EVA/MH composites is brittle and easily collapses into cracks, providing a channel for both heat transfer and oxygen, while suppling favorable conditions for the combustion of EVA. For EVA/MH-BF, due to the synergistic effect of BF on the carbon layer, the BF-reinforced carbon layer protects the EVA matrix under the flame, thereby slowing the heat transfer, oxygen transfer, and weakening the combustion intensity. Therefore, EVA/MH-BF further reduces the heat release and total smoke production, and exerts a synergistic smoke suppression effect.

### 3.3. Mechanical Property

**Figure 8:**
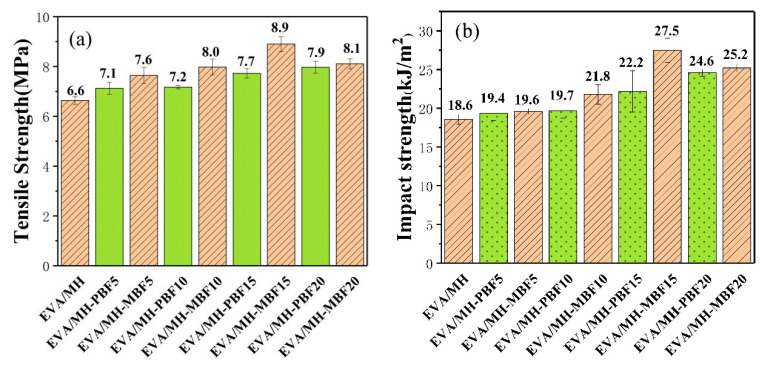
(**a**) Tensile strength, (**b**) non-notched impact strength of EVA/MH-BF composites.

**Figure 9:**
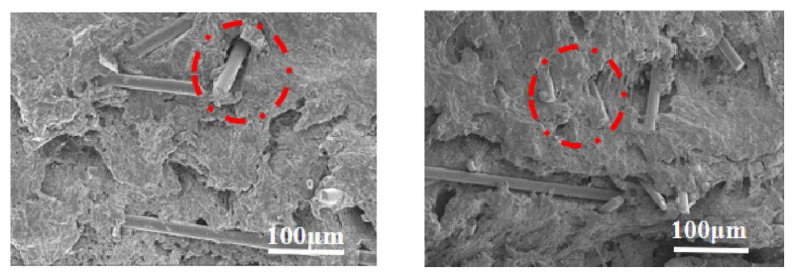
The SEM photos of EVA/MH-PBF15 and EVA/MH-MBF15 composites tensile fracture topography.

The mechanical properties of flame-retardant EVA composites are characterized by tensile and non-notched impact tests. Figure 8 shows the tensile strength and impact strength of samples EVA/MH, EVA/MH-PBF, and EVA/MH-MBF. Compared with EVA/MH, the addition of BF significantly improves the tensile strength and impact strength. Typically, the tensile strength of the composite EVA/MH-MBF15 is 8.9 MPa, and the impact strength is 27.5 kJ/m^2^, which is increased by 34.8% and 47.8%, respectively. In addition, we found that the mechanical properties of the EVA/MH- MBF composites showed a parabolic trend of first increasing and then decreasing, indicating that the addition of 15 phr MBF achieved the optimal index. When the amount of fiber added is higher than 15 phr, there may be factors such as agglomeration caused by excessive fiber content, which make the modification performance of the material drop back [29]. It is worth noting that both the tensile strength and impact strength of EVA/MH composites with MBF are higher than those of EVA/MH composites with PBF, which is due to the improved compatibility of the fiber with the EVA substrate. As shown in Figure 9, the voids between EVA matrix and MBF are significantly reduced, which helps the fibers to be better dispersed in the substrate.

**Figure 10:**
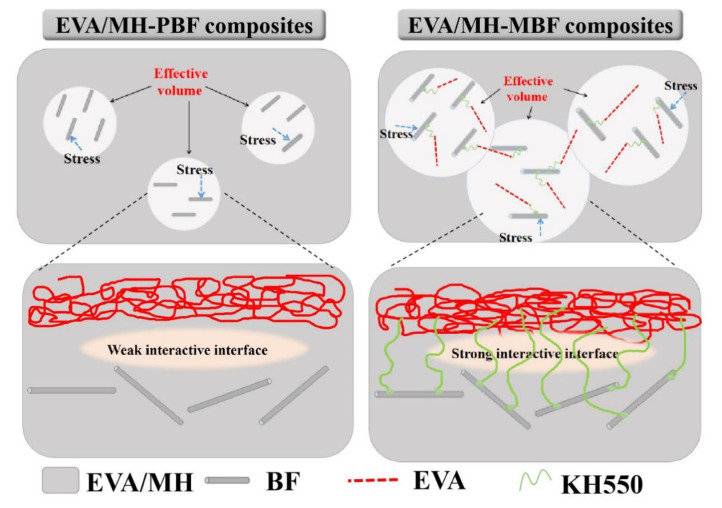
Schematic diagram of the force acting on composites.

According to the discussed results of the mechanical properties, the improvement in mechanical properties may be due to the following: (1) on introducing KH550 into the fiber surface, the interfacial interaction between the filler and the EVA matrix is improved, which makes the surface and matrix more tightly bonded [15,30], and as shown by the red dotted circle in Figure 9, the combination of MBF and the matrix material is tighter, and there is a gap between the PBF and the matrix material; (2) Due to its high specific surface area, the applied stress of the modified basalt fiber is readily transferred from the polymer matrix to the filler, thereby improving the mechanical properties of the material [31]. As shown in Figure 10, there should be a strong interface interaction between KH550 on the MBF surface and EVA/MH composites, increasing the effective volume of the composites accordingly. The expansion of the effective volume is presented by the continuous phase, which has a positive effect on adsorption and fixation for BF, and promotes the transfer of stress from the EVA matrix to the MBF. In contrast, because the interaction between PBF and the EVA matrix is weak, it cannot effectively increase the effective volume [32].

## 4. Conclusions

In summary, basalt fiber was successfully modified by KH550 via simple surface treatment, and further blended with EVA/MH composites. It was found that the overall flame retardant performances of EVA/MH-BF composites were maintained at a high level, and at the same time the mechanical properties were significantly improved. Compared with EVA/MH composites, the maximum mechanical properties can be improved by 34.8% and the impact properties by 47.8%. After the fiber surface modification, SEM showed that the MBF had better adhesion to the matrix than PBF, resulting in a better distribution of the fiber and significantly improved mechanical properties accordingly. Therefore, using KH550 basalt fiber for surface treatment of BF is a simple strategy to improve the mechanical properties of the EVA matrix.

## Figures and Tables

**Table 1:** Formulations of EVA/MH-BF Composites.

Sample	EVA/phr	MH/phr	PBF/phr	MBF/phr
EVA/MH	100	150	/	/
EVA/MH-PBF5	100	150	5	/
EVA/MH-PBF10	100	150	10	/
EVA/MH-PBF15	100	150	15	/
EVA/MH-PBF20	100	150	20	/
EVA/MH-MBF5	100	150	/	5
EVA/MH-MBF10	100	150	/	10
EVA/MH-MBF15	100	150	/	15
EVA/MH-MBF20	100	150	/	20

Note: “phr” means parts per hundreds of resin.

**Table 2:** CCT Data of EVA and EVA Composites.

	TTI (s)	pHRR (kW/m^2^)	TpHRR (s)	THR (MJ/m^2^)	pSPR (m^2^/s)	TSP (m^2^)	FIGRA (kW/(m^2^·s))
EVA	29 ± 4	1196 ± 271	175 ± 5	139 ± 15	0.100 ± 0.001	14.7 ± 0.7	6.83
EVA/MH	59 ± 6	260 ± 3	130 ± 25	109 ± 10	0.037 ± 0.002	10.1 ± 2.0	2.00
EVA/MH-PBF15	57 ± 1	242 ± 2	105 ± 5	96 ± 5	0.027 ± 0.002	8.4 ± 0.5	2.30
EVA/MH-MBF15	56 ± 1	219 ± 14	100 ± 15	93 ± 13	0.038 ± 0.009	8.6 ± 0.2	2.19
